# Inadequate conflict of interest policies at most French teaching hospitals: A survey and website analysis

**DOI:** 10.1371/journal.pone.0224193

**Published:** 2019-11-01

**Authors:** Christian Guy-Coichard, Gabriel Perraud, Anne Chailleu, Véronique Gaillac, Paul Scheffer, Barbara Mintzes

**Affiliations:** 1 AP-HP St Antoine Hospital, Paris, France; 2 Faculty of Medicine, Brest, France; 3 Chair of Formindep, Paris, France; 4 Hôpital Sainte-Anne, Paris, France; 5 Sciences of Education Department, Paris 8 University, Saint-Denis, France; 6 Faculty of Pharmacy, Charles Perkins Centre, The University of Sydney, Sydney, Australia; NIH/NCI/DCP/BRG, UNITED STATES

## Abstract

**Background:**

There are 32 teaching hospitals in France, including 30 University hospitals and two Regional teaching hospitals. Teaching hospitals have three roles: health care provision, training of healthcare professionals, and medical research. These roles lead to frequent interactions with pharmaceutical and medical device companies, inevitably raising risks of conflicts of interests. Therefore, policies to manage conflict of interests (COI) are crucial. This study aims to examine COI policies in French teaching hospitals.

**Methods:**

All French teaching hospitals (n = 32) were included in this study. All hospitals websites were screened for institutional COI policies and curriculum on COI, using standardized keyword searches. More data were collected through a questionnaire addressed to each chief executive officer (CEO) of the teaching hospital. We used predefined criteria (n = 20) inspired by similar surveys on COI policies in French, US and Canadian medical schools, with some additions to reflect the local hospital context. A global score for each hospital, ranging from 0 to 60 (higher scores denoting stronger policies) was calculated by summing points obtained for each criterion.

**Results:**

All 32 hospitals had websites; 21 hospitals listed policies or regulations on their websites or provided them on request. In December 2017, 17 (53.1%) had rules and regulations for some items only, four of which (12.5%) have considered implementing a policy, and only two (6.3%) have begun implementation. 15 (46.9%) had no evidence of COI policies and a null score. The maximum score was 24 out of 60.

**Conclusion:**

This is the first systematic assessment of COI policies in teaching hospitals in France. Such policies are needed to protect patients, clinicians and students from undue commercial influence. Despite public and political pressure for better management of COI, few teaching hospitals have implemented comprehensive and protective policies, and some hospitals lacked policies altogether. These results highlight the need for greater attention to management of COI within teaching hospitals. One potential solution would be to integrate COI policies into hospital accreditation procedures, in order to ensure a baseline of management at all teaching hospitals.

## Background

The rise of evidence-based medicine has been accompanied by increasing attention to commercial influences and conflicts of interests (COI). The more evidence there is to demonstrate the biases that could affect many aspects of medicine, the more it appears that these influences pose a risk to the health of patients.

In France, there are 32 teaching hospitals, which play a threefold role in training of nurses and physicians, medical research and medical care [1]. As key players in each of these functions, they interact extensively with healthcare industries. These interactions are extensive and varied, both at institutional and individual levels.

Teaching hospitals represent 37.9% of the country's public hospitals activity [1]. In their role as healthcare centers, hospitals are targeted in the promotion of health products. Hospitals are recognized to be at the peak of the prescription pyramid, with physicians in private practice tending to maintain patients on treatments initiated in hospital. The leverage effect of hospital prescriptions is therefore very important, especially for teaching hospitals that train future prescribers [2]: one hospital prescription may induce multiple prescriptions downstream. This effect, known as ‘the hospital to retail spillover’, has been analyzed and exploited by healthcare companies marketing and sales departments. [3, 4, 5, 6] In addition, a growing number of specialty products, generally amongst the most expensive, are reserved for hospital prescriptions.

Commercial promotion takes the well-known form of one-to-one sales representative visits, but also invitations (conferences, meals), sponsored education, support for publications and speaking engagements for opinion leaders, or even promotional events within the hospital premises [6–17]. According to the French national transparency database [18], 77.5% of physicians at teaching hospitals received at least one payment or gift (including in kind) over the period 2012–2016. In 2014, 84% of doctors registered with the National College of Physicians received payments or gifts declared in the database [19]. Medical faculty members are particularly sought-after by industry as key opinion leaders (KOL) who have a strong influence on the practices and representations of their present and future colleagues, and easy access to the media. [20–24]

At the institutional level, how hospitals are financed, especially research activities, introduces a conflict of interests. A growing proportion of teaching hospitals’ budgets, known as MERRI credits (Missions d’enseignement, de recherche, de référence et d’innovation -teaching, research, reference and innovation missions), is linked to criteria such as citation indices for publications, or the number of patients included in clinical trials [1]. At the same time, private companies are encouraged to outsource their research to public laboratories through an enhanced research tax credit. In this way, governments intend to align public research with private sector priorities in the hope of more immediate economic return. The creation of university or hospital foundations also makes it possible for the teaching hospital to be financed by healthcare firms, or even to create laboratories or teaching chairs benefiting from industrial sponsorship [1].

The legislation intended to prevent these many forms of influence within the teaching hospitals is the addition of measures taken over the course of decades. They tend to lack both comprehensiveness and specificity.

All French teaching hospitals are public institutions and the professionals who work there are civil servants. They are therefore subject to series of procedures and obligations aimed at ensuring integrity, neutrality and good governance of the public service by preventing conflicts of interests. The existing provisions, derived from the Code of Ethics for the Public Administration [25], cover, in particular, holding multiple jobs (be it in the public or private sector), and the law on public procurement. However, these legal provisions suffer from numerous shortcomings and exceptions.

The first French laws specific to health, known as “anti-gift laws”, were adopted a quarter of century ago (DMOS law of 27/01/1993). They have been progressively strengthened [26, 27], in particular to increase the transparency of links of interests between health professionals and healthcare institutions and economic players in the sector. These obligations were extended in particular after the benfluorex (Mediator) health scandal [28], which led, among other things, to the creation of a transparency register of interests inspired by the American Sunshine Act [18].

The Sales Visit Charter, a framework agreement between the Economic Committee for Health Products (CEPS), a governmental body, and the national association of the pharmaceutical industry (LEEM), sets out a set of rules for practice in the promotion of medicines to health professionals by pharmaceutical sales-representatives, agreed upon by all LEEM member companies [29].

In their role as centers of higher medical education, each teaching hospital is linked to a faculty of medicine through a contractual agreement. Policies for the prevention and management of COI in French medical schools were the subject of a previous study [30], which evaluated their scope and content, in line with initiatives previously carried out in the USA, Canada and Australia [31, 32, 33]. Following this study, the deans' conference adopted a voluntary Charter in 2017, which provides for cooperation with the CHUs in its implementation [34].

Finally, each teaching hospital has its own internal procedural rules, validated by the hospital Medical Commission, which can include more stringent measures to prevent conflicts of interests.

Contrary to the British National Health Service (NHS) [35], there is no comprehensive and uniform policy on conflicts of interests applicable to French university hospitals. Provision of health services within teaching hospitals is regulated by the Haute Autorité de Santé (High Authority for Health), but the law does not give it any real power to require policies to prevent COI, nor to monitor their implementation. Policies to prevent COI are not part of the criteria for accreditation of hospitals or health care institutions.

Therefore, we carried out this study in order to identify and evaluate existing conflicts of interests policies in the 32 teaching hospitals in France.

## Methods

### Scoring criteria for conflict of interest policies

This study is based on the criteria used in the AMSA Scorecard and by Shnier and al. [36, 32], and used in a previous study on COI policies in French medical schools [30]. These criteria were adapted to the hospital context in an AMSA study of 200 US teaching hospitals [37]. A working group of FORMINDEP, a French organisation that supports independent medical education, further adapted this scoring system to the French context, finalising the set of criteria used in this study.

The criteria take into account the specific activities of healthcare companies in teaching hospitals. In these criteria, "company" means any company involved in the field of health care, including medical devices and pharmaceuticals. We defined COI policies as any written, enforceable rule that is binding on all health professionals employed by the hospital.

In France, pharmaceutical companies may provide educational support to medical residents to assist them with publication of scientific articles. This can play an important role in a doctor's career. Moreover, continuing medical education (CME) may be hosted and organized by teaching hospitals. We therefore decided to include criteria to address both this publication assistance and continuing medical education (criterion 5) and, more broadly, all corporate support to physicians' careers (criteria 1, 3, 4, 5, 6).

Companies finance a large proportion of medical research and often have significant influence over the publication of results; we have included criteria for transparency of research and its funding (criteria 11,12).

The presence of company representatives within hospital departments is a key factor in the influence of firms and must be covered by agreements with the hospital (criteria 2, 8, 9).

Lastly, similarly to the approach taken by the AMSA Scorecard and Shnier et al., we also considered whether there were procedures in place for education, monitoring and enforcement (criteria 10, 13, 15, 16 17, 18, 19, 20).

We retained 20 criteria in total, which are listed in Box 1, 19 of which are rated incrementally from 0 to 3 (appendix 2):

- 0: the hospital has no policy for this criterion, or it is not accessible.
- 1: The hospital has an explicit policy, communicated to staff, but no additional information is available on regulatory and legal obligations for this criterion 1
- 2: the hospital policy is limited in scope, or monitoring is insufficient.
- 3: the hospital has an explicit policy on this issue at a high standard, reflecting standards in French law and contractual agreements between health authorities and health industry associations.

Scores for each of the criteria were summed to obtain a total score per teaching hospital. The maximum score per criterion was 3, leading to a total maximum score of 60. One criterion (#10) is binary in nature; we scored this 0 or 3 in order to avoid a lower weighting than the other 19 criteriaIn total, each hospital could obtain a maximum of 60 points. A detailed explanation of rating criteria is provided in S1 Table.

Legal obligations refer to the following laws: Laws 93–121 of 27/01/1993 ("Loi anti-cadeaux”, Anti-gifts Law), 2011–2012 of 29/12/2011 (Bertrand Law), 2016–41 of 26/01/2016 ("Loi de modernisation du système de santé”, Health System Modernization Law), the Public Health Code and the Medical Code of Ethics. [27]

**Box 1. Rating criteria**

1. Management of gifts and benefits
2. on site promotional presentations or speeches
3. participation in promotional events funded by companies
4. participation in medical conferences or internships funded by companies
5. accredited continuing education
6. ghostwriting
7. advisory or speaking activities on behalf of companies
8. access of representatives of pharmaceutical companies
9. access of representatives of medical equipment, biology and imaging companies
10. public disclosure of speaker’s interests
11. research funding
12. publication of clinical trials and transparency of research
13. hospital service associations
14. frameworks for market surveys
15. procurement of medicines and medical devices
16. conflict of interest education for teaching hospital staff
17. extension of the rules to all actors linked to the teaching hospital
18. governance rules
19. monitoring the application of rules and sanctions
20. authorities responsible for monitoring and reporting on conflicts of interest

### Data collection

We used several methods to obtain information on COI policies at the 32 teaching hospitals. First, we searched the website of each teaching hospital in May 2017 and November 2017 to find policies related to COI or documents interpreting policies using the French terms: « conflits d'intérêts » (conflict of interests), « liens d'intérêts » (competing interests), “declaration publique d’intérêts” (public declaration of interest), « industrie pharmaceutique » (pharmaceutical industry), « laboratoire pharmaceutique » (pharmaceutical firm), “éthique” or “deontologie” (ethics), “visiteurs médicaux” or “visite médicale” (companies representatives), “financement” (funding), « charte » (charter), et « règlement intérieur » (internal rules). The name of each policy and the latest date of adoption or the date of the policy’s most recent review were recorded.

Secondly, a registered letter (Appendices Files 1 and 2) was sent in May 2017 to each CEO of the 32 hospitals to inform them of the study. The letter explained its purpose, and the 20 criteria for which we required documentation. A second mail was sent in September 2017 to the 30 non-responding university hospitals. A third letter sent to the communications department requested information on the hospital’s internal rules and regulations.

At the same time, a letter was sent to the president of elected medical staff representatives of each hospital, to inform them about the study.

The hospital CEOs were informed that we were only interested in publicly available policies and that while respondents’ names would be kept confidential, the teaching hospitals and their policies would be identified in any subsequent publication. We mentioned that the study has the support of ANEMF (Association Nationale des Etudiants en Médecine de France–National Association of the French Medical Students) and ISNAR-IMG (InterSyndicale Nationale Autonome Représentative des Internes de Médecine Générale—National Autonomous Union of General Practitioners Interns).

Data collection continued until December 15, 2017.

Two reviewers independently scored each teaching hospital’s policies. Any reviewer employed by a teaching hospital was barred from scoring their own institution. The research team met and collectively reviewed ratings to ensure consistency of application and interpretation of criteria across reviewers. Any discrepancy between the two reviewers was addressed by the team, with any reviewers employed by a teaching hospital excluded from assessment of their establishment. Similarly to Shnier and al. [32], we summed the scores of the twenty individual categories for each hospital to come up with a global score, with a range of 0 to 58.

As no patients are involved in our study, and it relates to questions concerning policies at respondents’ institutions, rather than personal information, the Comité pour la Protection des Personnes (Comittee for human protection) Ile de France V, has written us that ethics approval or patient consent was not required for our research.

## Results

In December 2017, 13/32 teaching hospitals (40.6%) had rules and regulations for some items only, and another four (12.5%) have considered implementing a policy, two of which (6.3%) have begun implementation. Fifteen of the hospitals (46.9%) had no evidence of COI policies.

The 32 French teaching hospitals include 29 located in mainland France and 3 located in overseas territories. All of these university hospitals have a website. See S2 Table for a list of the university hospitals and their website.

In total, through mail, e-mail or screening of websites, we obtained information on 21 hospitals out of 32 (66%): Angers, Besançon, Bordeaux, Brest, Dijon, Lille, Limoges, Marseille (AP-HM), Montpellier, Nantes, Nîmes, Nice, Orléans, Paris (AP-HP), Poitiers, Reims, Rennes, Rouen, Saint-Etienne, Strasbourg, Toulouse. For the additional 11 hospitals for which no responses or information were obtained, we judged the hospital not to have a publicly-available COI policy as no indication of a COI policy was available via the hospital website.

### Mail and email survey

Eight hospital CEOs (18.6%), responded to our survey or sent us information on their internal regulations. These regulations were sometimes also available on-line.

Three CEOs (9%) responded directly to our first inquiry. There were: Assistance Publique—Hôpitaux de Paris (AP-HP), Toulouse, and Montpellier hospitals.The AP-HP provided us with publicly available reports about conflict of interests in their hospitals and detailed answers addressing our criteria. AP-HP set up a COI committee with the objective of assessing the status of conflict of interests within the institution and developing proposals for improvement. The commission submitted a report and proposals in May 2016, some of which have since been incorporated into internal regulations.

Toulouse hospital has installed a COI committee as of March 2017. Its main role is to develop a conflict of interest policy and evaluate its implementation. The hospital is working on this in close collaboration with the medical faculty of Toulouse.

Montpellier hospital is in the process of setting up a charter in order to improve their policies on COI, focusing on 10 elements. Although potentially addressing part of our criteria, we could not include these in our overview, as no charter had been published or sent to us by the survey end date.

We obtained a mail or e-mail response from five others teaching hopitals (15.6%): Angers, Besançon, Rennes, Rouen, Strasbourg. At the deadline of the study, the internal regulations of Besançon and Strasbourg were still under review. The internal regulations of Rennes and Rouen did not contain elements of a COI policy. Those of Angers contained some elements of a COI policy (Table 1).

**Table 1:** Overview of COI policy scores for each French teaching hospital (n = 17)*.

Hospital	Total score (range 0–608)	Score of 1 : policy exists, may not be active	Score of 2 : limited scope	Score of 3 : high standard
Toulouse	24	[1] management of gifts and benefits [2] promotional presentations or speeches [4] medical conferences funded by companies [8] [9] access of representatives [17] extension of rules to all actors in hospital	[7] advisory or speaking activities [13] hospital service associations [15] procurement of medicines or medical devices [16] COI education for teaching hospital staff [18] governance rules [19] monitoring the application of rules and sanctions	[14] frameworks of market surveys [20] authorities responsible for monitoring
APHP^1^	20	[2] promotional presentations [7] speaking activities [8] [9] access of representatives [17] extension of rules to all actors in hospital [19] monitoring the application of rules and sanctions	[1] management of gifts and benefits [13] hospital service associations [[15] procurement of medicines or medical devices [18] governance rules	[11], research funding [20] authorities responsible for monitoring
APHM^2^	12	[19] monitoring the application of rules and sanctions]	[1] management of gifts and benefits [7] advisory or speaking activities [17] extension of rules to all actors in hospital [18] governance rules	[15] procurement of medicines or medical devices
Montpellier	11		[1] management of gifts and benefits [7] advisory or speaking activities [8] [9] access of representatives	[14] frameworks of market surveys
Poitiers	9	[8] [9] access of representatives	[7] advisory or speaking activities [15] procurement of medicines or medical devices	[3] promotional events funded by companies
Angers	8		[1] management of gifts and benefits [7] advisory or speaking activities [8] [9] access of representatives	
Nantes	6	[1] management of gifts and benefits [1,11]	[7] advisory or speaking activities [15] procurement of medicines or medical devices	
Reims	6	[2] promotional presentations or speeches [15] procurement of medicines or medical devices	[1] management of gifts and benefits [7] advisory or speaking activities	
Lille	4	[7] advisory or speaking activities		[1] management of gifts and benefits
Nîmes	4		[1] management of gifts and benefits [7] advisory or speaking activities	
Nice	3	[16] COI education for teaching hospital staff [17] extension of rules to all actors in hospital [20] authorities responsible for monitoring		
Orléans	3	[7] advisory or speaking activities	[1] management of gifts and benefits	
Rouen	2		[1] management of gifts and benefits	
Besançon	1	[1] management of gifts and benefits		
Brest	1	[11] research funding		
Dijon	1	[1] management of gifts and benefits		
Bordeaux	1	[7] advisory or speaking activities		

* An additional 15 French teaching hospital had no information on conflict of interest policies on their websites, and failed to reply to our request for information

^1^ Assistance Publique—Hôpitaux de Paris

^2^ Assistance Publique—Hôpitaux de Marseille

(See S2 Table for the list of all French teaching hospitals).

### Internet screening

We also screened the websites of the 32 teaching hospitals, 13 (40.6%) of which had additional information on COI policies on their websites. Appendix 3 provides a list of the hospitals and information sources.

### University hospital scores

By December 15 2017, only two hospitals have implemented a comprehensive COI policy (Toulouse and AP-HP Paris), and another two have initiated of policies that are not yet implemented (Montpellier and AP-HM Marseille).

We applied our 20-criteria scoring scale [range 0–60] to the 32 hospitals: 15 (46,9%) have a zero score, 13 (40,6%) a score of less than 10, 4 (12,5%) a score of 10 to 24. Toulouse hospital had the maximum score of 24. AP-HP (Paris) had a score of 20, AP-HM (Marseille) a score of 12, Montpellier a score of 11, Poitiers 9, Angers 8, Nantes and Reims 6, Lille and Nîmes 4, Nice and Orléans 3, Rouen 2, and Besançon, Bordeaux and Dijon 1.

The criteria with the highest scores were management of gifts and benefits, with two hospitals, Lille and Poitiers, having policies judged to be at a high standard, procurement of medicines and medical devices, one hospital, AP-HP, with a policy at a high standard, and monitoring and reporting of COI (AP-HP, high standard). Frameworks for market surveys were at a high standard in two hospitals, Montpellier and Toulouse. Eight additional hospitals policies were limited in scope. Table 1 provides an overview of hospital scores.

All hospitals scored zero on five criteria, indicating that these were no COI policies covering these activities: participation of hospital staff in promotional events funded by companies, industry-sponsored accredited continuing education, ghostwriting, public disclosure of speakers’ financial interests, and policies requiring publication of clinical trials carried out at the hospital.

## Discussion

This is the first published study of its kind in France, on teaching hospitals’ COI policies. Our study highlights mixed results and, in general, limited attention to COI policies at French teaching hospitals. Of the 32 hospitals, only 4 have taken explicit steps towards developing and implementing COI policies, and in two cases these policies are still under development. These policies are also incomplete, missing important activities that can lead to conflicts in teaching hospitals. On the other hand, more than half of hospitals (17 over 32) have elements of COI management policy, but the approach taken is often limited in scope and/or lacks mechanisms for implementation.

Certain criteria received a high score in several hospitals, reflecting important steps towards independence from corporate influence. These included adaptations to recent laws in France, for example on gifts and benefits, speaking activities, procurement and medical services, and creation of an commitee for monitoring COI. Other major criteria appear not to have been considered, even in the hospitals with the most comprehensive COI policies. These included participation of medical staff in promotional events, industry-sponsored accredited continuing education, ghostwriting, public disclosure of financial interests, and clinical trial publication and research transparency.

Media coverage of a similar survey on COI policies in French medical schools carried out in 2017 [30] had a major impact on subsequent policy, leading the medical faculties deans' conference to draft a charter to address conflicts of interests [34]. The natural links between teaching hospitals and faculties (even if they depend on different administrations) may have encouraged some hospitals to begin to reflect and develop actions in this field as well. The top ranking hospital, Toulouse, developed its policy after the publication of the survey of medical faculties, in cooperation with the medical faculty. On the other hand, APHP hospitals (Paris) had begun to discuss implementation of COI policies prior to the study publication. AS compared with medical faculties, teaching hospitals have a wider range of scores, which range from 0 to 24 out of 60 points,. This suggests that awareness of the need to manage COI is still heavily dependent on the local context and actors. A national initiative involving all teaching hospitals in France could lead to a more consistent approach to addressing COI.

We also found that in a few cases, teaching hospitals had begun to consider development of COI policies, but had not yet implemented them, an encouraging step in terms of future policy development. Among others, Toulouse hospital has a regularly updated work plan and set up an ethics committee. APHP has set up a commission devoted to the prevention of conflicts of interest, whose report served as a basis for the first amendments to the internal regulations, and the creation of a foundation for research. Montpellier has developed a charter containing ten commitments to prevent conflicts of interest, particularly in terms of transparency, relations with companies, governance, transparency in research and the appointment of an ethics officer. However, this charter had not yet been implemented when we completed our survey. These are promising developments, reflecting a real dynamic for change, not yet fully represented in these study results.

Transparency of research funding and of clinical trial results, in particular requirements for publication (criteria 11,12,13) are important elements of policy development, as teaching hospitals have an important role in research and these policies aim to mitigate publication bias. However, the registration and publication of clinical trials is not addressed by any teaching hospital. Eight French teaching hospitals were amongst the 20 sponsors internationally with the largest rate of unreported trials registered on clinicaltrials.gov, according to the Alltrials initiative [38].

Several hospitals have set up foundations to centralize external funding of their research. However, a score of 3, reflecting a high policy standard, required the publication of amounts received from companies, and that the foundation replaces and eliminates prior funding circuits, namely ad hoc charities created and managed by the hospital departments, known as ‘associations de service’. These are prone to opacity and conflicts of interests and the French General Accounting Office has recommended their elimination [1]. Even the APHP and Toulouse hospitals, who had the most comprehensive COI policies among our sample, have not effectively tackled this issue yet. Strong opposition from some key opinion leaders and the Contract Research Organizations and Biotech sectors had already led the government to water down these regulations and allow hospitals to maintain the ‘associations de service’ [39, 40].

Education on conflicts of interest, which is now being developed in some medical schools, is still absent from hospitals: none reached a score above zero. There is place for significant, rapid and easy improvement in this area.

Finally, the best COI prevention policy cannot be sustainable without explicit processes in place for monitoring and sanctions; only two teaching hospitals have so far set up committees with the responsibility to monitor compliance and issue sanctions, APHP and Toulouse.

We have little evidence of international comparisons, since only the American Medical Students Association (AMSA) has carried out a similar study in 200 teaching hospitals in the United States in 2014. Unfortunately, the results have not been published, but an unpublished report is available on the AMSA archived website [37]. This study, carried out in 2014, found, among 204 teaching hospitals, a "perfect score" (grade 3) in 90% of hospitals for enforcement of policies, in 65% of cases for medical device representatives, between 40 and 50% of cases for ghostwriting, gifts, consulting, meals, and speakers bureaus; poor scores are obtained for medical sales representatives, disclosure of COI, COI education. Even if it is difficult to compare two very different educational and hospital systems, it appears that the work carried out over the years by AMSA on this subject has made it possible to achieve progress in the existence and use of COI policies, which encourages the same work in France.

Furthermore, the dynamics triggered by studies on medical schools seems to have a similar impact on teaching hospitals in the United States and in France.

### On the relevance of our analysis grid and rating criteria, and the methodological limitations of our survey

This is the first study of its kind in Europe for teaching hospitals. The rating scale that we used is adapted from a scale used for medical faculties. There are difference in the scope of activities within teaching hopitals, and the scale will certainly require further methodological corrections, even if our methodology is largely based on a list of significant clinical [21, 41, 42, 43, 44, 45] or institutional [19, 29, 35, 46, 47, 48, 49, 50] references. The rating given in the appendix (our definition of ratings) is a proposal for interpretation, to be adapted according to the realities of the hospital.

A number of the criteria in this scale address activities that occur commonly in teaching hospitals: receipt of gifts or benefits, disclosure of interests, the creation of monitoring or sanctioning bodies. While studies of medical faculties focus on initial medical training, our study required integration of the influence of companies on continuing professional education, on the clinical trials that are carried out in hospitals, on ancillary activities of doctors and other health professionals, and on sales representatives’ access to hospital premises including clinical services.

The definition of ratings provided in the appendix is a proposal for interpretation, to be adapted according to the realities of the hospital setting. Criteria within this ordinal scale are applied in an incremental manner, with each higher rating assuming that the criteria for lower ratings have been fulfilled.

Some of these criteria, built *de novo*, may need to be corrected in future studies. For example, we have noted that several of the hospitals internal rules include a reminder of the civil service code provisions prohibiting all healthcare personnel from receiving any benefit or rewards, or from engaging in paid work outside the hospital. This regulation allows derogations only for doctors, allowing them to carry out activities on behalf of healthcare companies (consulting, expertise, teaching). When the hospital regulation does not specify any waiver, we had to consider that any advantage or ancillary activity is indeed prohibited and grant a rating of 2 or 3 for criteria 1 and 7. However, experience and data from the public transparency database prove that this policy is not enforced [18]. These criteria definitions shall warrant revision in future studies.

Public disclosure of interests is addressed in three of the criteria: whether clinical staff working within a hospital must declare their COI (criterion 10).; whether staff involved in procurement and managerial positions must declare COI (criteria 15 and 18). We retained the three criteria because of the different nature of these functions and because internal hospital rules often differentiate between these functions.

We maintained criteria that achieved a null score in all hospitals because these criteria address activities that occur in teaching hospitals and are major components of COI policy. These are the criteria for participation in promotional events or conferences funded by companies, funding for continuing education, ghostwriting, public declaration of interest by any external stakeholder, publication of clinical trials and transparency of research, and teaching on conflict of interest.

Due to the low response rate to our three requests for information, our findings rely mostly on charters and internal rules and regulations published online. This may introduce a discrepancy between hospitals based on the availability or not of the corresponding documents online. But we considered that websites are an important source of information for hospital staff and patients, and that documents concerning COI management should be publicly available there.

It is also important to note that publication of the internal rules and regulations are a mandatory, public document by law (Code de la santé publique L6143-1). Their absence from the hospital website or the refusal to grant access to it upon a freedom of information access request is an indication of poor policy in itself.

We noted that hospitals with a developed policy had communicated publicly about their initiative in the media. It is therefore unlikely that a hospital with a substantial policy went unnoticed in this study.

It is hoped that the next survey, if necessary, will enable hospitals to correct data they consider insufficient. The lessons learnt from AMSA faculties studies clearly demonstrate the dynamics one can expect from such a ranking.

## Conclusion

This is the first survey in France to examine COI policies at teaching hospitals. Despite the laws and regulations, the public interest and media coverage, too few hospitals have taken steps to develop a real policy that protects patients and caregivers. However, some effort was made locally, to draft or implement such a policy, even if the result is incomplete at the moment. There is no national policy driven by the public authorities; examples of implementation are mainly the result of individual initiatives by Chief Executives, which have been highlighted in the media. Health services can and must be proactive in reducing corporate influence[51], but it is essential that each public hospital structure implement a strong conflict of interest management policy, models of which are now publicly available, some of which incorporate most of the criteria we have used [52]. In France, teaching hospitals do not face any centralised mandate or legal imperative to implement conflict of interest policies. Instead, decisions about whether and how to implement such policies are made separately by each institution. Our findings strongly suggest that reliance on spontaneous individual institutional decision-making results in inadequate, piecemeal and incomplete governance, with many commonly-encountered conflicts of interest not addressed. This is despite the benfluorex (Mediator) safety scandal of 2009–2010, which led to intense attention to the role of conflicts of interest in health care and to legislative changes.

This study shows that spontaneous regulation, carried out individually by each institution, has resulted in limited oversight of COI. Public authorities should consider introducing COI policy as part of the accreditation procedures for hospitals.

We note that similar studies in the USA and France, concerning medical schools, have encouraged advances in oversight of COI. The scores of American faculties increased conctantly over time, following repeated surveys by the American Medical Students’Association (AMSA), whih were based on a scorecard [53]. Professional asociations have also taken a stand on the subject of COI [54]. The conference of French medical schools deans took a strong position on the subject after the French study on faculties [34]. These observations can reinforce the need for such studies at regular intervals.

## Supporting information

S1 Table Scoring system for the 20 items of the study. (DOCX)

S2 Table French teaching hospitals websites table. (DOCX)

S1 File Letter to the CEOs of the teaching hospitals, French. (DOCX)

S2 File Letter to the CEOs of the teaching hospitals, English. (DOCX)
