# Supplementary material for: Inadequate conflict of interest policies at most French teaching hospitals: A survey and website analysis
Source: PLoS One. 2019 Nov 1;14(11):e0224193. doi: 10.1371/journal.pone.0224193 (PMC6824557; doi:10.1371/journal.pone.0224193)
Supplement: S1 Table — (DOCX) [file pone.0224193.s001.docx]

**Appendix table 1 : Guide for the scoring of the 20 criteria**

All criteria (except Nr10) are rated from 0 to 3:

Note 0: the hospital has no COI prevention policy for this criterion, or it is not accessible.

Note 1: The hospital has an explicit policy of prevention of COI, communicated to staff, but this policy does not provide any additional information in relation to regulatory and legal obligations for this criterion.

Note 2: the hospital explicit policy for COI meets limited requirements, or their monitoring is insufficient.

Note 3: the hospital explicit policy for COIs meets a high level of requirements.

**Criterion 1: Management of gifts and benefits**

Note 3: Any benefit financed by a company, regardless of its nature or value, including catering during staff or convivial moments, is prohibited.

Note 2: Business financing of the following benefits is prohibited:

"benefits directly related to training (manuals, brochures, materials, flyers,),

The term "catering" is used to refer to the provision of catering, which is provided during a continuing education course funded by the company or provided on site through direct or indirect financing by the company.

Note 1: The hospital has an explicit policy of preventing CIs, communicated to staff, which requires only the publication of any advantage financed by a company.

**Criterion 2: Presentations or Promotional Speeches**

Note 3: Promotional presentations or speeches are forbidden within the hospital premises.

Note 2: Promotional presentations or speeches are tolerated only at non-validating, non-mandatory and unpaid meetings.

Note 1: The hospital has an explicit policy for the prevention of CoI, communicated to staff, which authorizes presentations and promotional speeches, only outside healthcare departments.

**Criterion 3: Participation in promotional events funded by companies.**

Note 3: Participation in events funded entirely by corporate sponsor sis prohibited.

Note 2: Participation in an event funded by a corporate sponsor, provided that the participant is neither financially supported nor remunerated, is tolerated.

Note 1: The hospital has an explicit CoI prevention policy in this context, communicated to staff, which does not limit participation in an event funded by a company.

**Criterion 4: Attendance at medical conferences or internships funded by companies.**

Note 3: Participation in conferences or trainings funded by health care companies is prohibited for all hospital professionals, including staff in training.

Note 2: Participation in a conference or training funded by a company is permitted, provided that the participant is neither financially supported nor remunerated.

Note 1: The hospital has an explicit policy for the prevention of CoIs in this context, communicated to staff, but this does not limit participation in a conference or training funded by a company.

**Criterion 5: Accredited Continuing Medical Education (CME)**

Note 3: Hospital staff are not allowed to participate in CME activities funded, directly or indirectly, by healthcare companies.

Note 2: Hospital staff are authorized to participate in CME funded, directly or indirectly, by health care companies, provided the participants bear a part of the cost of the program.

Note 1: The hospital has an explicit policy for the prevention of COIs in this case, communicated to staff, but this does not limit participation in corporate sponsored CME activities

**Criterion 6: Ghostwriting**

Note 3: The involvement of all authors, including medical writer, is presented in the publications signed by hospital staff. The writing of articles or publications as a "ghost " author (ghostwriting), or the signature as an honorary author, is prohibited.

Note 2: The involvement of all authors, including medical writer, is presented in the publications signed by hospital staff.

Note 1: The hospital has an explicit policy of preventing COIs in this context, communicated to staff, but this does not limit participation in publications.

**Criterion 7: advisory or speaking activities on behalf of companies**

Note 3: Consultancy for commercial or marketing purposes are prohibited. Activities for scientific purposes are subject to authorization by the hospital.

Note 2: All ancillary activities are subject to authorization by the hospital.

Note 1: The hospital has an explicit policy for the prevention of COIs in this context, communicated to staff, but this does not limit the activities of consultants and speakers.

**Criterion 8: Access by representatives of pharmaceutical companies**

Note 3: Sales representatives do not have access to medical departments within the hospital.

Note 2: Sales representatives can access medical departments, provided they have a formal agreement with the hospital, which prohibits contacts outside referents designated by the hospital structure.

Note 1: The hospital has an explicit policy for the prevention of COIs in this context, communicated to staff, which allows presentations by representatives of pharmaceutical companies only under the form of collective meetings.

**Criterion 9: Access of representatives of medical equipment, biology and imaging companies**

Note 3: Sales representatives of medical, biological or imaging equipment companies may access hospital services only for non-commercial reasons, such as technical assistance or equipment training.

Note 2: The presence of sales representatives of medical device companies is allowed for commercial reasons, but they may only be in contact with the referents designated by the hospital structure.

Note 1: The hospital has an explicit policy for the prevention of COIs, communicated to staff, which allows presentations by representatives of medical devices companies only under the form of collective meetings.

**Criterion 10: Public disclosure of speaker’s interests**

Note 0: No conflict of interest prevention policy.

Note 3: The hospital explicit policy of prevention of COIs requires all speakers (external or personal) within the hospital to disclose their interests.

**Criterion 11: Research Funding**

Note 3: The hospital has set up a compulsory centralized collection system (e. g. a hospital foundation) for private funding of medical research.

Note 2: The list of sponsors participating in the financing of medical research involving the hospital teams, and the amount of funding, are public.

Note 1: The hospital has an explicit policy for the prevention of COI in this context, communicated to staff, but this does not require the publication of private funding for medical research involving the hospital staff.

**Criterion 12: Publication of clinical trials, transparency of research**

Note 3: In addition to the criteria in Note 2, the hospital policy includes an open data policy for clinical trials sponsored by the hospital and encourages the opening of data from trials in which it participates.

Note 2: The hospital policy and contracts with a third-party sponsor require that all trials in which the hospital participates be registered and a summary of the results posted on at least one primary registry approved by WHO. Any confidentiality clause in industrial sponsorship contracts must explicitly exclude from its scope information obligations owed to participating patients and pharmacovigilance obligations.

Note 1: It is the hospital policy that all trials sponsored by the sponsoring hospital should be registered prior to entry of the first patient, and an abstract of the results published within 12 months of the end of the trial, on at least one primary registry approved by WHO.

**Criterion 13: Hospital service associations**

Note 3: Associations operating in hospitals, whether or not headquartered within the premises, are not allowed to raise funds from healthcare companies. The authorization to operate care, research or teaching activities is subject to the domiciliation of accounts with the public accountant. The hospital has set up a centralized (e. g. a Foundation) and transparent channel for the collection of private funding, whatever its destination.

Note 2: The hospital policy states that associations operating in hospitals must first be registered on a public list and authorized. Associations having the hospital as registered address should have their accounts managed by the public accountant.

Note 1: The hospital has an explicit policy of preventing COIs in this context, communicated to staff, but this policy does not provide any additional information in relation to regulatory and legal obligations for this criterion.

**Criterion 14: Framework for market surveys**

Note 3: The hospital CoI prevention policy specifically prohibits staff participation in market surveys conducted by healthcare companies.

Note 2: The hospital CoI prevention policy specifically prohibits staff participation in market surveys where remunerated. Note 1: The hospital has an explicit policy of preventing COIs in this context, communicated to staff, but this does not limit participation in market surveys.

**Criterion 15: Procurement of medicines and medical devices**

Note 3: Participation in purchasing and evaluation decisions for medical products, devices or equipment is prohibited for personnel having a link of interest with a stakeholder. Note 2: Any hospital personnel involved in purchasing and evaluating medical products, devices or equipment must submit a public declaration of interest.

Note 1: The hospital has an explicit policy for the prevention of COIs in this context, communicated to staff, but it does not require a public disclosure of interests from staff involved in purchasing and evaluating medicines.

**Criterion 16: Conflict of interest education for teaching hospital staff**

Note 3: Issues related to conflicts of interests in medical research, clinical practice and the training of health professionals are regularly included in the continuing education of the hospital staff.

Note 2: A training session on the issues related to conflicts of interest in medical research, clinical practice and the training of health professionals is organized for the hospital staff.

Note 1: The hospital has an explicit awareness policy on the prevention of COIs, communicated to staff, but it does not include training on conflicts of interest.

**Criterion 17: Extension of the rules to to all actors linked to the teaching hospital**

Note 3: The COI prevention policy applies to all the hospital personnel, regardless of their status, and in all places where they perform their professional tasks, even if the local establishment does not have the same requirements.

Note 2: The COI prevention policy applies in only one of the two situations above.

Note 1: The hospital has an explicit policy for the prevention of COIs, communicated to staff, but this does not specify that it applies to staff in all settings.

**Criterion 18: Governance rules**

Note 3: All personnel involved in the hospital governance is barred from participating in decisions where he/she has a competing interest. Note 2: All personnel involved in the hospital governance must submit a regularly updated public disclosure of interests.

Note 1: The hospital has an explicit policy for the prevention of COIs, communicated to staff, but this policy does not require public disclosure of interest or restrictions in participation.

**Criterion 19: Monitoring the application of rules and sanctions**

Note 3: The policy implementation is monitored and non-compliance subject to effective sanctions. An annual review is carried out and made public.

Note 2: At least one of the above actions is carried out.

Note 1: The hospital has an explicit policy for the prevention of COIs, communicated to staff, but this does not include any monitoring of its enforcement.

**Criterion 20: authorities responsible for monitoring and reporting on conflicts of interest**

Note 3: The hospital has appointed a deontologist or an ethics committee to monitor, inform and advise on the prevention of COIs.

Note 2: The hospital has appointed a deontologist, or deontology commission, to provide information and advice on the prevention of COIs.

Note 1: The hospital has an explicit policy for the prevention of COIs, communicated to staff, but it does not designate a dedicated deontologist or ethics committee.
