# Supplementary material for: Inadequate conflict of interest policies at most French teaching hospitals: A survey and website analysis
Source: PLoS One. 2019 Nov 1;14(11):e0224193. doi: 10.1371/journal.pone.0224193 (PMC6824557; doi:10.1371/journal.pone.0224193)
Supplement: S2 Table — (DOCX) [file pone.0224193.s002.docx]

| **Teaching Hospital** | **Website address** |
| --- | --- |
| Amiens | <http://www.chu-amiens.fr/> |
| Angers | <http://www.chu-angers.fr/> |
| AP-HM Marseille | <http://www.ap-hm.fr/> |
| AP-HP Paris | <http://www.aphp.fr/> |
| Besançon | <http://www.chu-besancon.fr/> |
| Bordeaux | <http://www.chu-bordeaux.fr/> |
| Brest | <http://www.chu-brest.fr/> |
| Caen | <http://www.chu-caen.fr/> |
| Clermont-ferrand | <http://www.chu-amiens.fr/> |
| Dijon | <http://www.chu-dijon.fr/> |
| Grenoble | <http://www.chu-grenoble.fr/> |
| Guadeloupe | <http://www.chu-guadeloupe.fr/> |
| La réunion | <http://www.chu-reunion.fr/> |
| Lille | <http://www.chu-lille.fr/> |
| Limoges | <http://www.chu-limoges.fr/> |
| Lyon | <http://www.chu-lyon.fr/> |
| Martinique | <http://www.chu-fortdefrance.fr/> |
| Metz-Thionville | <http://www.chr-metz-thionville.fr/> |
| Montpellier | <http://www.chu-montpellier.fr/> |
| Nancy | <http://www.chru-nancy.fr/> |
| Nantes | <http://www.chu-nantes.fr/> |
| Nice | <http://www.chu-nice.fr/> |
| Nîmes | <http://www.chu-nimes.fr/> |
| Orléans | <http://www.chr-orleans.fr/> |
| Poitiers | <http://www.chu-poitiers.fr/> |
| Reims | <http://www.chu-reims.fr/> |
| Rennes | <http://www.chu-rennes.fr/> |
| Rouen | <http://www.chu-rouen.fr/> |
| St Etienne | <http://www.chu-st-etienne.fr/> |
| Strasbourg | <http://www.chu-strasbourg.fr/> |
| Toulouse | <http://www.chu-toulouse.fr/> |
| Tours | <http://www.chu-tours.fr/> |
