# Supplementary material for: Inadequate conflict of interest policies at most French teaching hospitals: A survey and website analysis
Source: PLoS One. 2019 Nov 1;14(11):e0224193. doi: 10.1371/journal.pone.0224193 (PMC6824557; doi:10.1371/journal.pone.0224193)
Supplement: S1 File — (DOCX) [file pone.0224193.s003.docx]

Paris, le 2 mai 2017

## Formindep

**www.formindep.org**

Madame la Directrice Générale du CHU, Monsieur le Directeur Général du CHU,

Nous dirigeons une étude ayant pour objet les politiques de prévention des conflits d’intérêts des Centres Hospitalo-universitaires français. Notre méthodologie se base sur les travaux similaires ayant conduit depuis 2007 aux classements des facultés de médecine américaines^[[1]](#footnote-1)^, canadiennes^[[2]](#footnote-2)^, et australiennes^[[3]](#footnote-3)^, puis des CHU américains, et enfin des facultés de médecine françaises en 2016 ^4^. Notre étude est financée par l’association FORMINDEP (association loi 1901 qui milite pour une formation et une information des professionnels de santé indépendante des entreprises), et soutenue par l’ANEMF.

L’objectif de cette étude est d’évaluer les moyens mis en œuvre par les CHU pour répondre à l’exigence éthique de prévention des influences indues sur les soins, la recherche et l’enseignement. Si le travail commun avec les industriels pharmaceutiques est une nécessité, il ne doit jamais placer le prescripteur ou le professionnel de santé en situation de contrainte ou d’influence, ni interférer avec l’intérêt du patient.

Les enquêtes menées dans les pays anglo-saxons ont poussé les facultés et CHU à développer un ensemble d'initiatives de formation et de protection concernant les conflits d'intérêts. L’American Association of the Medical Colleges qui représente 145 facultés de médecine états-uniennes, 17 facultés de médecine canadiennes et plus de 400 hôpitaux universitaires a pris des positions fortes dès 2008 sur le sujet.^[[4]](#footnote-4)^5 Les résultats positifs en termes de qualité de prescription et de soins découlant de ces nouveaux enseignements font déjà l'objet de publications dans les revues médicales de référence pour ce qui concerne les changements opérés dans les facultés.^[[5]](#footnote-5)^6

Cette dynamique est d'ailleurs renforcée par la prise de position publique de certains officiels. Suite à la parution dans la revue PLOS One de notre étude évaluant les politiques de prévention des conflits d’intérêts au sein des facultés de médecine^^[[6]](#footnote-6)^^, la Conférence des doyens des facultés de médecine avait confirmé par un communiqué de presse le 16 janvier 2017 les lacunes des politiques actuelles, et regretté qu’“*une partie des relations avec l’industrie [soit] essentiellement due à la politique menée au sein des services hospitaliers*”.

Notre recherche devrait ainsi permettre de se faire une idée précise de la dynamique correspondante en France pour les CHU, qui n'a fait à ce jour l'objet d'aucune étude spécifique.

Si vous avez des politiques définies, en cours d'élaboration ou à l'état de projet, nous vous serions également reconnaissants de nous indiquer leur calendrier de finalisation, et de nous les communiquer dès que possible. Vous pouvez retourner votre réponse par courrier (à l’adresse ci-dessous), ou par mail ([classement.chu@gmail.com](mailto:classement.chu@gmail.com)).

Les politiques de votre établissement seront analysées selon les vingt catégories suivantes : avantages, présentations ou discours promotionnels, participation à des événements, des conférences ou des stages financés par les firmes, financement de la formation continue, ghostwriting, publication des essais cliniques, activités de conseil et d’orateur, accès des représentants des entreprises pharmaceutiques ou de matériel médical, encadrement du démarchage, déclarations publiques d’intérêts des personnels intervenants, et des personnels participant aux décisions de gouvernance, enseignements relatifs aux conflits d'intérêts et à l'influence des firmes du médicament et des dispositifs médicaux, financements de la recherche par l'industrie, associations de service, achats de médicaments et dispositifs médicaux, extension des règles à tous les acteurs en lien avec le CHU, instances de surveillance des conflits d’intérêts. Vous trouverez ci-joint le détail de ces critères ainsi que la cotation qui pourra conduire à un classement des 32 CHU français.

Nous prendrons de nouveau contact avec vous après avoir effectué l'analyse des informations que vous nous aurez communiquées, afin de vous donner la possibilité de confirmer l'exactitude de nos résultats et de notre description de la politique de votre établissement concernant les conflits d'intérêts.

Étant donné que nous ne demandons que les documents publics relatifs à ces politiques, l'information en découlant ne demeurera pas confidentielle, et les CHU seront identifiés par leur nom. Vous pouvez, bien entendu, refuser de participer ou vous retirer du projet à tout moment. Cependant, toutes les politiques publiques seront retenues même si l'un des Directeurs de CHU contactés se retire.

Pour tout renseignement complémentaire, n'hésitez pas à nous contacter par le biais de l'adresse mail mentionnée précédemment.

Nous vous remercions d'avance pour l'aide que vous pourrez apporter dans la réalisation de cette étude importante pour la qualité des soins prodigués aux patients, et de la formation des personnels hospitaliers.

Dans l'attente de votre réponse, nous vous prions de recevoir nos salutations les plus distinguées.

Pour le FORMINDEP

La Présidente, Anne CHAILLEU

Les Directeurs de l’étude :

Christian GUY-COICHARD

Jean-Sébastien BORDE

1. http://www.amsascorecard.org/ [↑](#footnote-ref-1)
2. Shnier A, Lexchin J, Mintzes B, Jutel A, Holloway K (2013) Too Few, Too Weak: Conflict of Interest Policies at Canadian Medical Schools. PLoS ONE 8(7): e68633 [↑](#footnote-ref-2)
3. Mason P., Tattersall M.H.N. (2011), Conflicts of interest : a review of institutional policy in Australian medical schools, The Medical Journal of Australia ; 194 : 121-125

   4 Scheffer P, Guy-Coichard C, Outh-Gauer D, Calet-Froissart Z, Boursier M, Mintzes B, et al.(2017) Conflict of Interest Policies at French Medical Schools: Starting from the Bottom. PLoS ONE 12(1): e0168258. doi:10.1371/journal.pone.0168258 [↑](#footnote-ref-3)
4. 5 Les facultés de médecine et les hôpitaux universitaires devraient concevoir des standards de curriculum ainsi que des ressources pédagogiques pour toutes les phases de la formation médicale - de la formation initiale comprenant l'internat, à la formation continue – procurant des outils pour former les étudiants et les membres des facultés sur les procédés et les disciplines impliquées dans la découverte, le développement, les tests cliniques, la sécurité, la valeur thérapeutique et la régulation des médicaments. American Association of Medical Colleges. *IndustryFunding of Medical Education: Report of an AAMC Task Force.* 2008 [↑](#footnote-ref-4)
5. 6 King M. et al., “Medical school gift restriction policies and physician prescribing of newly marketed psychotropic medications” BMJ 2013;346:f264 [↑](#footnote-ref-5)
6. [↑](#footnote-ref-6)
