# Supplementary material for: Inadequate conflict of interest policies at most French teaching hospitals: A survey and website analysis
Source: PLoS One. 2019 Nov 1;14(11):e0224193. doi: 10.1371/journal.pone.0224193 (PMC6824557; doi:10.1371/journal.pone.0224193)
Supplement: S2 File — (DOCX) [file pone.0224193.s004.docx]

Paris, May 2, 2017

## Formindep

**www.formindep.org**

Madam or Mr CEO of the teaching hospital of …,

We are leading a study on the conflict of interest prevention policies of French Teaching Hospitals. Our methodology is based on similar work that has led since 2007 to the rankings of American^[[1]](#footnote-1)^, Canadian^[[2]](#footnote-2)^and Australian^[[3]](#footnote-3)^ medical schools, then American teaching hospitals, and finally French medical schools in 2016. ^4^ Our study is funded by the FORMINDEP association (an association under the 1901 law that promotes training and information for health professionals independent of companies), and supported by the ANEMF.

The objective of this study is to evaluate the means implemented by teaching hospitals to meet the ethical requirement of preventing undue influences on care, research and teaching. While working together with pharmaceutical companies is a necessity, it must never place the prescriber or health professional in a situation of constraint or influence, nor interfere with the patient's interest.

Surveys conducted in Anglo-Saxon countries have prompted faculties and teaching hospitals to develop a range of training and protection initiatives regarding conflicts of interest. The American Association of the Medical Colleges, which represents 145 American medical schools, 17 Canadian medical schools and more than 400 teaching hospitals, has taken strong positions on the subject since 2008. ^[[4]](#footnote-4)^5 The positive results in terms of quality of prescription and care resulting from these new teachings are already published in the medical journals of reference with regard to the changes made in the faculties. ^[[5]](#footnote-5)^6

This dynamic is reinforced by the public stance of some officials. Following the publication in PLOS One of our study assessing policies to prevent conflicts of interest within medical schools^^[[6]](#footnote-6)^^, the Conference of Deans of Medicine confirmed in a press release on 16 January 2017 the shortcomings of current policies, and regretted that "*part of the relationship with industry[is] essentially due to the policy pursued within hospital services*".

Our research should thus provide a precise idea of the corresponding dynamics in France for teaching hospitals, which has not yet been the subject of any specific study.

If you have policies defined, under development or in draft form, we would also appreciate it if you could let us know when they will be finalized and provide us with them as soon as possible. You can return your answer by mail (to the address below), or by email [(](mailto:classement.chu@gmail.com)classement.chu@gmail.com).

Your institution's policies will be analyzed according to the following twenty categories: benefits, presentations or promotional speeches, participation in events, conferences or internships financed by the firms, financing of continuing training, ghostwriting, publication of clinical trials, consulting and speaking activities, access for representatives of pharmaceutical or medical device companies, supervision of canvassing, public declarations of interest of the personnel involved, and personnel involved in governance decisions, lessons relating to conflicts of interest and the influence of drug and medical device companies, research funding by industry, service associations, drug and medical device purchases, extension of the rules to all stakeholders in connection with the University Hospital, conflict of interest monitoring bodies. You will find attached the details of these criteria as well as the rating that could lead to a ranking of the 32 French teaching hospitals.

We will contact you again after having analysed the information you have provided us, in order to give you the opportunity to confirm the accuracy of our results and our description of your institution's conflict of interest policy.

Since we only request public documents related to these policies, the information resulting from them will not remain confidential, and THs will be identified by name. You can, of course, refuse to participate or withdraw from the project at any time. However, all public policies will be retained even if one of the TH Directors contacted withdraws.

For any further information, do not hesitate to contact us via the e-mail address mentioned above.

We thank you in advance for your support in carrying out this study, which is important for the quality of patient care and the training of hospital staff.

We look forward to hearing from you and look forward to receiving our best regards.

For FORMINDEP

The President, Anne CHAILLEU

The Study Directors:

Christian GUY-COICHARD

Jean-Sébastien BORDE

1. http://www.amsascorecard.org/ [↑](#footnote-ref-1)
2. Shnier A, Lexchin J, Mintzes B, Jutel A, Holloway K (2013) Too Few, Too Weak: Conflict of Interest Policies at Canadian Medical Schools. PLoS ONE 8(7): e68633 [↑](#footnote-ref-2)
3. Mason P., Tattersall M.H.N. (2011), Conflicts of interest : a review of institutional policy in Australian medical schools, The Medical Journal of Australia ; 194 : 121-125

   4 Scheffer P, Guy-Coichard C, Outh-Gauer D, Calet-Froissart Z, Boursier M, Mintzes B, et al.(2017) Conflict of Interest Policies at French Medical Schools: Starting from the Bottom. PLoS ONE 12(1): e0168258. doi:10.1371/journal.pone.0168258 [↑](#footnote-ref-3)
4. 5 Medical schools and teaching hospitals should develop curriculum standards and educational resources for all phases of medical education - from initial training including internship, to continuing education - providing tools to train students and faculty members on the processes and disciplines involved in drug discovery, development, clinical testing, safety, therapeutic value and regulation. American Association of Medical Colleges. *IndustryFunding of Medical Education: Report of an AAMC Task Force.* 2008 [↑](#footnote-ref-4)
5. 6 King M. et al., “Medical school gift restriction policies and physician prescribing of newly marketed psychotropic medications” BMJ 2013;346:f264 [↑](#footnote-ref-5)
6. [↑](#footnote-ref-6)
